# Ex Vivo Anatomical Characterization of Handsewn or Stapled Jejunocecal Anastomosis in Horses by Computed Tomography Scan

**DOI:** 10.1155/2014/234738

**Published:** 2014-12-03

**Authors:** Gessica Giusto, Bryan Iotti, Federica Sammartano, Alberto Valazza, Marco Gandini

**Affiliations:** Department of Veterinary Sciences, University of Turin, Largo Braccini 2-5, 10095 Grugliasco, Italy

## Abstract

The aim of this study is to compare handsewn and stapled jejunocecal anastomosis with different stomal lengths in terms of anatomical differences. Group 1 underwent a two-layer handsewn jejunocecal side-to-side anastomosis (HS); Group 2 received a stapled jejunocecal side-to-side anastomosis (GIA). Each group was divided into two subgroups (HS80 and HS100, GIA80 and GIA100). Specimens were inflated and CT scanned. The stomal/jejunal area ratio and blind end pouch volume/area were measured and compared. Effective length of the stoma was measured and compared with the initial length. Stomal/jejunal area ratio was 1.1 for both 80 techniques, 1.6 for the GIA100, and 1.9 for the HS100 technique. Both HS and GIA techniques produced a blind end pouch and exhibited a mean increase of the final stomal length ranging from 6 to 11% greater than the original stomal length. All techniques will exhibit a length increase of the final stomal length compared to the intended stomal length, with a consequent increase in stomal area. Stapled techniques consistently produced a large distal blind end pouch. Length of a jejunocecal anastomosis should be selected in accordance with the diameter of afferent jejunum, and the 80 mm stomal length could be deemed sufficient in horses.

## 1. Introduction

Jejunocecal anastomoses with resection are commonly performed in equine abdominal surgery whenever the ileum is damaged to such an extent that will not allow performing an end-to-end anastomosis. Although end-to-side anastomosis was considered as the original technique [1], side-to-side techniques can have fewer complications and offer a better prognosis [2]. Nevertheless, the complication rates of these techniques are still high while survival rates are lower when compared to end-to-end jejunojejunal anastomosis for both handsewn and stapled techniques [3, 4].

Possible explanations have been proposed mostly from a functional point of view, related to the peculiarity that this anastomosis joins two segments with very specific and different physiology and motility patterns. The overcoming of intracecal pressure by the jejunum [5] without the coordination normally produced by the ileocecal valve [2, 6] and the fact that most of the proximal jejunum has already been distended and possibly damaged by the primary pathology [7] are the main factors that could explain the poor performance of this type of anastomosis, both handsewn and stapled. Furthermore, it is still being debated whether a handsewn or stapled technique should be preferred in order to reduce complications and improve survival rates [3, 4]. This and other factors could give some mechanical contribution to proposed functional problems [8].

There is a strong emphasis on finding ways of improving the technique [9], but in the last 30 years not much has been done towards this goal for either handsewn or stapled anastomosis. Although considered more technically demanding and involving more procedures when compared to other types of anastomosis [1], jejunocecal anastomosis could also be more sensitive to minor changes [3].

Previous literature [3] suggested that stomal dimensions can play a role in the development of postoperative complications.

Our hypothesis is that anatomical factors that could contribute to the success or the failure of jejunocecal anastomosis in horses do exist and possibly explain differences in outcome between stapled and handsewn techniques.

Thus, the aim of this study is to compare handsewn and stapled jejunocecal anastomosis with different stomal lengths in terms of anatomical characterization.

## 2. Materials and Methods

Intestinal specimens comprising the cecum, ileum, and three meters of jejunum from 24 horses (mean age 24 months, range 18–30 months, mean weight 400 kg, and range 380–430) were collected immediately after death at an abattoir.

Bowel segments were divided into two groups: Group 1 (HS) underwent a two-layer handsewn jejunocecal side-to-side anastomosis with lactomer 9-1 2-0 suture (Polysorb, Covidien Italia, Milano) while Group 2 (GIA) received a stapled jejunocecal side-to-side anastomosis performed with a linear cutting stapler (Autosuture Multifire GIA80 and GIA100, Covidien Italia, Milano). Each group was divided into two subgroups (HS80 and HS100, GIA80 and GIA100), on the basis of the intended length of the initial stoma (80 or 100 mm) or the length of the stapler used (for the stapled techniques), that is, 80 or 100 mm for the GIA80 group and the GIA100 group, respectively. Surgical techniques for two layers handsewn and stapled jejunocecal anastomosis were performed as previously described [10, 11]. After completion of the anastomosis, bowel segments were then inflated to a pressure of 8 mmHg and submitted to CT scanning [11]. This pressure was selected in a preliminary study as the pressure that would maximally distend the stoma without damage to the intestinal suture edges [12].

### 2.1. Image Acquisition, Multiplanar Reconstructions, and Stomal Area, Proximal Jejunal Area, Blind End Pouch Volume, and Distal Jejunal Area Measurements

**Figure 1:**
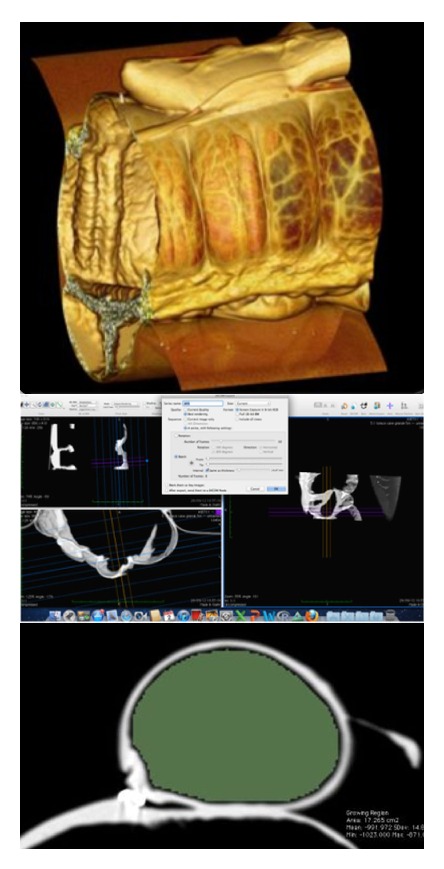
Top: 3D reconstruction of a handsewn jejunocecal anastomosis; middle: MPR reconstruction of a handsewn jejunocecal anastomosis; bottom: MPR reconstruction and calculation of the area of the afferent jejunum.

The image acquisition and reconstruction, as well as the data gathering, were performed as previously described [11] (Figure 1).

Briefly, the samples were acquired using a single slice computed tomography unit (GE High Speed FX/I, General Electric, Fairfield, CT, USA,) in axial mode using a slice thickness of 1 mm, a matrix size of 512 × 512, a medium smooth reconstruction algorithm, 120 KVp, and 130 mAs. The images were transferred to the visualization workstation (Apple iMac, 21.5′′ Mid 2011, 2.5 Ghz i5, 8 GB RAM, Mac OS X 10.7.5, http://www.apple.com) and viewed with standardized windowing parameters (WindowsWidth 970, WindowsLevel −414.50). An image-processing software (Osirix 4.1.2 32-bit, http://www.osirix-viewer.com) was then used to calculate the volume and area of the blind end pouch. Three-dimensional MPR of the stoma was exported as DICOM lossless images, for use in image measurement software (ImageJ 1.47d 64-bit, http://rsb.info.nih.gov/ij/). Six stapled (GIA) stomas were not monoplanar and required the use of thick-slice MPR reconstructions using volume rendering (MPR slice thickness range: 8.19 mm to 38.12 mm, mean ± SD: 19.06 ± 11.87). Using the wand tool (tolerance 100, legacy mode) the stomal area and perimeter were calculated on the MPR images, while the proximal jejunal area and perimeter were calculated on the original images.

### 2.2. Stomal/Intestinal Area Ratio

In each specimen, a ratio was obtained by dividing the stomal area by the afferent jejunal area measured 2 cm proximal to the stoma.

### 2.3. Blind End Pouch Volume/Distal Jejunal Area Ratio

Because the volume of the blind end pouch is given by the length of the pouch and by its area, to avoid bias given by different small intestinal areas, we obtained an aspect ratio by dividing the volume of the blind end pouch measured as described above by the area calculated on the first slice distal to the stoma.

### 2.4. Percentage of Increase in Stomal Length

The percentage of increase was obtained by comparing the length of the stoma, measured directly with a caliper on the specimen at the time of incision and after deflation following image acquisition. For handsewn techniques the initial length of the stoma was standardized with a ruler when making the incisions on the jejunum and the cecal wall, while for the stapled techniques the length of the cut performed by the in-built blade of the stapler was considered (e.g., 80 or 100 mm). After the CT scan was completed, the cecum was opened and the effective length of the stoma was measured with a caliper and compared to the initial length.

The percentage of increase was obtained with the formula increase % = (*L*_*f*_ − *L*_*i*_)/*L*_*i*_∗100 where *L*_*i*_ is the initial length and *L*_*f*_ is the effective length of the stoma.

### 2.5. Statistical Analysis

Using a commercial statistical software (Instat, Graphpad, La Jolla, CA, USA) we measured the normality of each group using a Kolmogorov-Smirnov normality test.

If data were normally distributed, results were reported as mean ± SD, while if data were not normally distributed, the results were reported as median (min–max value). The value of *P* for statistical significance was set at *P* < 0.05.

We used unpaired *t*-test on normally distributed data and Mann-Whitney Test on nonnormally distributed data. We compared handsewn or stapled techniques with different initial length and then handsewn against stapled techniques of the same length.

## 3. Results

### 3.1. Stomal/Intestinal Area Ratio

The mean (±SD) ratio was 1.183 ± 0.212 for the HS80 and 1.93 ± 0.924 for the HS100 and the difference was not statistically significant (unpaired *t*-test Welch corrected, *P* = 0.1107).

The difference of ratio between the GIA80 group (1.1 ± 0.172) and the GIA100 group (1.643 ± 0.34) was statistically significant (unpaired *t*-test Welch corrected, *P* = 0.0101).

Comparing different techniques of the same length (HS80 versus GIA80 and HS100 versus GIA100) did not show any significance (unpaired *t*-test Welch corrected, *P* = 0.4749 and *P* = 0.4988, resp.).

### 3.2. Difference in Blind End Pouch Volume and Area Ratio

The difference in ratio of the HS80 group (0.64 ± 0.172) and of the HS100 (1.051 ± 0.57) group was nonsignificant (unpaired *t*-test Welch corrected, *P* = 0.2038).

The median ratio of the GIA80 group was 1.242 (1.020–2.307) and was not significantly different compared to the median value of 2.04 (1.252–2.845) of the GIA100 group (Mann-Whitney, *P* = 0.0649).

Comparing the two techniques (handsewn versus stapled) showed significant difference when the intended length of the stoma was either 80 (Mann-Whitney, *P* = 0.0043) or 100 mm (unpaired *t*-test Welch corrected, *P* = 0.0215).

### 3.3. Percentage of Increase in Stomal Length

For all comparisons, a Mann-Whitney test was used. The percentage of increase in stomal length compared to intended length was 11.5 (2–20) for HS80 group and 9 (1–20) for HS100 group. The difference is not statistically significant (*P* = 0.4704).

For the GIA80 group the percentage of increase was 8.5 (6–14) and for the GIA100 group it was 5 (5–9). The difference was not quite significant (*P* = 0.649).

Comparing the HS80 group with the GIA80 group, the difference was not significant (*P* = 0.574) as in the case of the comparison between the HS100 and GIA100 groups (*P* = 0.3768).

## 4. Discussion and Conclusions

A jejunocecal side-to-side anastomosis connects two bowel segments with very different shapes and physiology but, nevertheless, aims to restore the physical and physiological continuity of the resected bowel. The less physical and physiological obstacles are encountered by the ingesta at the level of the anastomosis, the better it will work.

Stomal dimensions could play a critical role in the outcome of jejunocecal anastomosis in horses. Despite the fact that some authors [13] advocate the formation of a large stoma to avoid stenosis and anastomosis blockage, Freeman stated that the stoma “*should be close in size to the diameter of the jejunum feeding into it to optimize function of a jejunocecostomy*” [14]. He reported a survival rate higher than 90% by producing, with the handsewn technique, a stomal size close to the jejunum proximal to the anastomosis [3].

In our study the ratio of stomal and intestinal areas was obviously influenced by the initial length of the stoma. In stapled anastomosis we found a significant difference between the 80 or 100 mm length, with the latter producing a stomal area more than 1.5-fold the area of the afferent jejunum. Also in the handsewn group the 100 mm long incision produced a stoma of area nearly double that of the afferent jejunum, although the difference with the 80 mm long stomas was not significant. One of the possible mechanisms to explain the higher complication rates of jejunocecal anastomosis compared to other techniques is the possibility that this technique allows a reflux of cecal content into the jejunum [5]. This is probably directly related to stomal dimensions. A 100 mm long incision (or employing a 100 mm long linear cutting stapler) produces a stomal area between 1.5 and two times the intestinal area. This leads to the formation of a large passage in the cecal wall that could, in our opinion, favor reflux into the jejunum proximal to the anastomosis. This could be caused by the fact that the jejunum must overcome the intracecal pressure without the controlling mechanism of the ileocecal valve [2, 5, 6]. A paper by L. M. Srivastava and V. P. Srivastava [15] extends previous work by Shapiro et al. [16] regarding peristalsis and fluid dynamics by formulating a mathematical model which allows the precise calculation of the pressure rise occurring inside a circular nonuniform tube with a sinusoidal wave traveling down its wall and filled with a non-Newtonian fluid exhibiting asymmetric flow. The jejunum can be considered to follow this mathematical model. If the stomal area is significantly greater than the jejunal area, the pressure rise, intended as the increase in pressure exerted on the fluid inside the nonuniform tube, originating from the jejunum towards the cecum, is much smaller than if the stomal/intestinal area ratio is close to one. One portion of the generated pressure must be lost to filling the larger surface area of the distal part of the diverging tube (it diffuses over a larger area) and cannot be employed in aiding its axial progression [15]. This behavior underlines the importance of properly selecting the desired stomal length in order to produce a sufficient but not excessive stomal area. A stomal area that is inferior to the area of the jejunum that leads to it would instead result in a greater pressure rise but might hinder the free flow of solid ingesta. A stomal/intestinal area ratio close or slightly inferior to 1 will therefore aid the jejunum in resisting the backpressure experienced from the cecum, despite the lack of an ileocecal valve. As an example this is what happens by placing a thumb on the extremity of a garden hose: reducing the area increases the pressure at which the fluid exits the hose. It should however be noted that the above-mentioned model considers a non-Newtonian fluid with rheological flow behavior indexes ranging from 0.33 to 1 [15]. The model does not consider the effects of the suspension of solid objects (fragments of feed) or of a mucous layer (as it may affect the nonslip condition) [17] and assumes a sinusoidal peristaltic pattern. In a paper from Freeman and Schaeffer [3], the authors reported a case of handsewn jejunocecal anastomosis that they performed with a stomal length of 7-8 cm and that was subject to a necroscopy nineteen months later, to be found with a stoma of about 15 cm in length and a distended proximal small intestine. In the same paper Freeman and Schaeffer reported stomal enlargement and small intestinal dilation also for a stapled case. The authors considered the stomal and distal jejunal dilation to be the cause of the distension of the small intestine. Furthermore, Edwards [18] reported a case in which the stoma of a jejunocecal side-to-side anastomosis reduced to 2 cm diameter but did not impede the flow of ingesta, although possibly reducing cecal reflux. This is completely in accordance with our theory that a larger stoma increases the difficulty for the distal jejunum in overcoming cecal pressures.

A blind end pouch consistently formed with the examined techniques, although with significantly higher volume/area ratio in the GIA anastomosis. This is probably related to the fact that in the HS technique there is the possibility for the surgeon to reduce the length of the blind end, while in the GIA technique the presence of the beveled plastic tip causes consistent formation of a blind end pouch of 16–20 mm, as previously described [11]. Presence of a blind pouch could affect outcome and complication rates in clinical settings [1, 3, 10, 11], and surgeons must be aware that while it is possible to reduce it with perfect technique in handsewn anastomosis, for stapled anastomosis, the stapler itself or the technique must be modified [11].

With both techniques, we measured a mean increase of the final stomal length compared to the intended stomal length. Surgeons must take into account that any technique and length will exhibit an increase between 6 and 12% of the final stomal length compared to the intended stomal length, with a consequent increase in stomal area and all the previously mentioned consequences.

Cecal body wall conformation could also play a role in the formation of the stoma. Placing the stoma on a haustrum or between two haustra could modify stomal shape. We standardized the position of the stoma by placing it about 20 cm from the ileocecal valve and approximately midway between the dorsal and medial cecal band, but we did not take into consideration the position relative to the haustra. Described techniques advocate the placement of the stoma on a cecal sacculation: we found that the sacculations seen on an empty cecum do not always correspond to true haustra or sacculations of a mildly distended organ.

During our work we also noticed that stapled stomas were not monoplanar but resulted in a spindle shape stoma curved dorsoventrally. The double row of staples typical of GIA techniques appears to behave similarly to a bicycle chain, allowing a high degree of movement dorsoventrally but not laterolaterally. The staple line exhibits a higher degree of rigidity compared to a handsewn row of stitches and because of this the two sides of the stomal incision can only bend dorsoventrally. Separation is made possible by the short terminal part of handsewn suturing that, by acting as a pivot point, allows the staple lines to rotate, while it could be restrained by the perimeter of the small intestine. A larger jejunal perimeter allows a higher degree of separation of the sides of the incision and results in a larger stomal area. This probably does not occur in GIA100 because the longer incision already produces a larger stomal area without requiring a high degree of separation.

Despite our best efforts to standardize the position of the stoma on the cecal wall as much as possible, we must assume that there have been variations between samples. The position of the stoma in relation to the cecal tenia could also have influenced its shape and margin separation.

As a limit of our study, these anatomical analyses on a freshly performed anastomosis do not take into account inflammation and tissue healing that could alter the conformation of the stoma in the postoperative period.

To reduce interoperator variables, the same surgeon Marco Gandini performed all the anastomosis, but obviously not in blind fashion. Also the measurement on intestinal samples and on CT scan images has been performed by the same operator Bryan Iotti but again not in a blind modality (staples would show off in CT scan images, making samples identifiable). We could not find a method to perform this study in a blind fashion, and this could be a limit of the study itself.

Based on our results, performing a jejunocecal anastomosis in 400 kg horses with an initial stoma length of 80 mm is sufficient to produce a stoma as wide as the jejunum proximal to it, regardless of the technique used and taking into account that the final length will be increased up to 12%.

While with the handsewn technique the surgeon can reduce the formation of a blind end pouch by making the incisions in the intestines close to the distal end of the jejunal stump, with stapled techniques the formation of a blind end pouch is consistent and cannot be avoided without modifying the anvil of the stapler or modifying the technique.

This work carries the limitations of an ex vivo study and further in vivo examinations are needed before applying these results in a clinical setting.

## Abbreviations

CT: Computer tomography
DICOM: Digital imaging and communication in medicine
*L*_*f*_: Effective length of the stoma of the anastomosis
*L*_*i*_: Initial length of the stoma of the anastomosis
MPR: Multiplanar reconstruction.

## References

[1] Freeman D. E. (1997). Surgery of the small intestine. *The Veterinary Clinics of North America. Equine Practice*.

[2] Rocken M., Ross M. W. (1989). Vergleichsstudie uber die jejunocaecostomie als end-zu-seitanastomose und seit-zu-seitanastomose. *Pferdeheilkunde*.

[3] Freeman D. E., Schaeffer D. J. (2010). Comparison of complications and long-term survival rates following hand-sewn versus stapled side-to-side jejunocecostomy in horses with colic. *Journal of the American Veterinary Medical Association*.

[4] Proudman C. J., Edwards G. B., Barnes J. (2007). Differential survival in horses requiring end-to-end jejunojejunal anastomosis compared to those requiring side-to-side jejunocaecal anastomosis. *Equine Veterinary Journal*.

[5] Huskamp B. (1973). Ileum resection and jejunocecostomy in the horse. *Berliner und Munchener Tierarztliche Wochenschrift*.

[6] Roger T., Malbert C. H. (1989). Caracteristiques anatomo-fonctionelles de la jonction ileocecale du poney. *Revue de Médecine Vétérinaire*.

[7] Dabareiner R. M., Sullins K. E., Snyder J. R., White N. A., Gardner I. A. (1993). Evaluation of the microcirculation of the equine small intestine after intraluminal distention and subsequent decompression. *The American Journal of Veterinary Research*.

[8] Freeman D. E., Hammock P., Baker G. J. (2000). Short- and long-term survival and prevalence of postoperative ileus after small intestinal surgery in the horse. *Equine Veterinary Journal. Supplement*.

[9] Freeman D. E., Schaeffer D. J. (2005). Short-term survival after surgery for epiploic foramen entrapment compared with other strangulating diseases of the small intestine in horses. *Equine Veterinary Journal*.

[10] Freeman D. E., White N. A., Mair T. S., Moore J. N. (2008). Surgical techniques. *The Equine Acute Abdomen*.

[11] Gandini M., Giusto G., Sammartano F. (2014). In vitro description of a new technique for stapled side-to-side jejunocecal anastomosis in horses and CT scan anatomical comparison with other techniques. *BMC Veterinary Research*.

[12] Giusto G., Amedeo S., Gandini M. (2014). Effects of staple size, tissue thickness, and precompression time on staple shape in side-to-side jejunocecal anastomosis in specimens obtained from healthy horses at an abattoir. *American Journal of Veterinary Research*.

[13] Edwards G. B. (1986). Resection and anastomosis of small intestine: current methods applicable to the horse. *Equine Veterinary Journal*.

[14] Freeman D. E. Jejunocecostomy—technique and tips to success.

[15] Srivastava L. M., Srivastava V. P. (1985). Peristaltic transport of a non-Newtonian fluid: applications to the vas deferens and small intestine. *Annals of Biomedical Engineering*.

[16] Shapiro A. H., Jaffrin M. Y., Weinberg S. L. (1969). Peristaltic pumping with long wavelength at low Reynolds number. *Journal of Fluid Mechanics*.

[17] Ferrua M. J., Singh R. P. (2010). Modeling the fluid dynamics in a human stomach to gain insight of food digestion. *Journal of Food Science*.

[18] Edwards G. B. (1981). Obstruction of the ileum in the horse: a report of 27 clinical cases. *Equine Veterinary Journal*.

